# Cavitary lesions emerged rapidly in *Pseudomonas aeruginosa* pneumonia

**DOI:** 10.1002/ccr3.2704

**Published:** 2020-02-13

**Authors:** Naoki Kawakami, Shin Ohara, Ho Namkoong

**Affiliations:** ^1^ Department of Emergency and Critical Care Medicine St. Luke's International Hospital Tokyo Japan; ^2^ Department of Hematology Eiju General Hospital Tokyo Japan; ^3^ Department of Pulmonary Medicine Eiju General Hospital Tokyo Japan; ^4^ Laboratory of Clinical Immunology and Microbiology National Institute of Allergy and Infectious Diseases MD USA

**Keywords:** cavitary lesions, community‐acquired pneumonia, lung abscesses, *Pseudomonas aeruginosa* pneumonia

## Abstract

*Pseudomonas aeruginosa* should be highly considered as a causative pathogen, when patients deteriorate rapidly despite community‐acquired pathogen, and the radiological findings display a rapid emergence of cavitary lung lesions especially among patients at high risk of *P aeruginosa* pneumonia.

## Graphical abstract

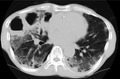

## 1 PICTURE IN CLINICAL MEDICINE

A 59‐year‐old male ex‐smoker who received chemotherapy for lung adenocarcinoma a month ago presented with one‐week history of shortness of breath without any antibiotics. As risk factors for pseudomonas colonization or infection, he had a recent history of chemotherapy and underlying lung diseases of lung cancer and COPD.

**Figure 1:**
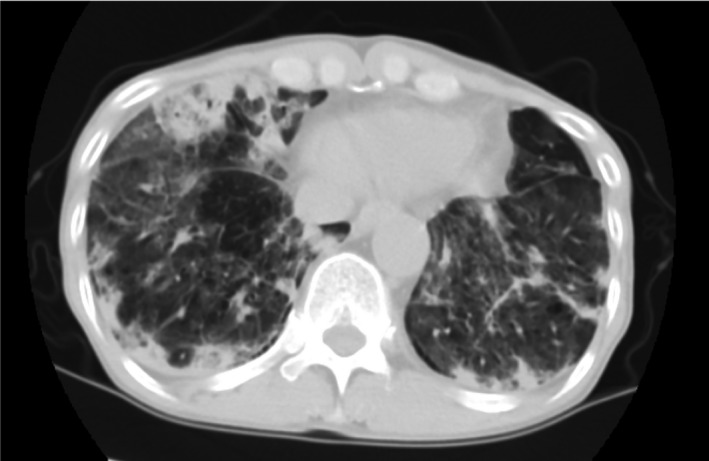
Chest computed tomography scan obtained at admission, showing consolidation and ground‐glass opacity in both the lower lung lobes

**Figure 2:**
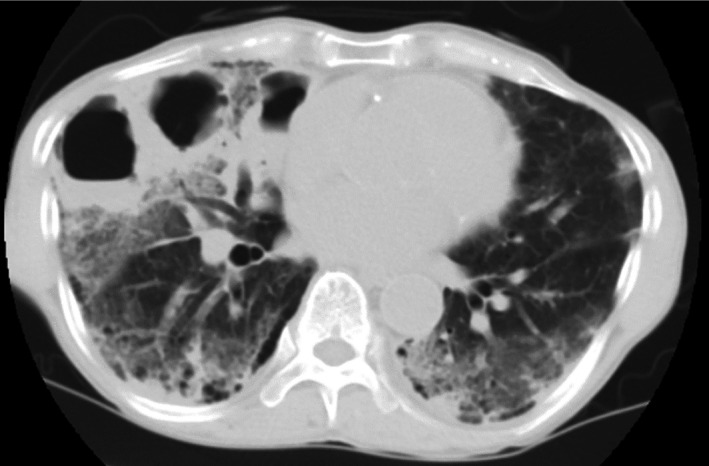
Chest computed tomography scan obtained three days after admission, showing multiple giant cavitary lesions with air‐fluid levels in the right lower lung lobe

Chest computed tomography (CT) revealed consolidation and ground‐glass opacities in bilateral lower lobes (Figure 1). We initiated antibiotics for presumptive community‐acquired pneumonia (CAP), but his respiratory condition worsened after 3 days of treatment. A repeat CT revealed multiple rapidly emerging giant cavitary lesions with air‐fluid levels in right lower lobe (Figure 2). He died the next day. Sputum and fluid cultured from cavitary lesions during autopsy were positive for *Pseudomonas aeruginosa*.

*Pseudomonas aeruginosa* is a common pathogen of nosocomial pneumonia but rare in CAP. It is the etiological agent in 0.9%‐1.9% of patients with CAP requiring hospitalization. However, the mortality rate with CAP due to *Pseudomonas aeruginosa* is reported to be 61.1%.^1^ It occasionally induces rapid and progressive tissue destruction, leading to the formation of cavitary lesions and abscesses.^2^ When CAP patients display unusual rapid growth of cavitary lesions, *P aeruginosa* should be strongly considered as a causative pathogen.

## CONFLICT OF INTEREST

None declared.

## AUTHOR CONTRIBUTIONS

All authors participated in the review of the manuscript. NK: drafted the manuscript. SO and HN: participated in the data collections. HN: drafted the manuscript. All authors read and approved the final manuscript.
